# Intergenerational Tutoring: Older Adults Supporting Children’s Academic Needs via Video Conferencing

**DOI:** 10.1093/geroni/igab046.3407

**Published:** 2021-12-17

**Authors:** Jessica Hoffman, Edward Miller, Jeffrey Burr, Jan Mutchler, James Hermelbracht, Robert Volpe, Robin Codding, Amy Briesch

**Affiliations:** 1 Northeastern University, Boston, Massachusetts, United States; 2 University of Massachusetts Boston, Boston, Massachusetts, United States; 3 McCormack Graduate School, Boston, Massachusetts, United States; 4 University of Massachusetts Boston, University of Massachusetts Boston, Massachusetts, United States; 5 Northeastern University, Northeastern University, Minnesota, United States; 6 Northeastern University, Northeastern University, Massachusetts, United States

## Abstract

COVID-19 resulted in societal disruptions across the lifespan. School (K-12) closures were among the most challenging impacts of the virus, leaving many parents with the burden of schooling their children at home. Another major impact of the virus was the social isolation and loneliness felt by many retired, older adults, who were sheltering at home. The disruptions of COVID-19 led our inter-professional team to develop the Intergenerational Tutoring program. Intergenerational Tutoring addresses a service delivery gap in schools because tutors expand schools' capacity to implement evidence-based instruction with students in need of individual support. At the same time, research shows that meaningful volunteering supports the well-being of older adults across physical, psychosocial and cognitive dimensions of health. The aim of the Intergenerational Tutoring program is to pair older adults with kindergarten children in high needs schools to implement early literacy interventions remotely via Zoom. Our poster will describe the Intergenerational Tutoring program including tutor training and tutoring implementation. We will summarize the initial findings from our pilot study conducted in spring and summer 2021 with tutors and children. Data will include (1) themes from tutor interviews regarding the personal meaningfulness of the program and the program’s associated benefits and challenges; (2) implementation fidelity data; (3) impact of tutoring on children’s early literacy skills; and (4) parent feedback. We will summarize lessons learned and next steps for the program.

